# Commentary: Pivoting during a pandemic: developing a new recruitment model for a randomised controlled trial in response to COVID-19

**DOI:** 10.1186/s13063-021-05567-0

**Published:** 2021-09-08

**Authors:** Shakira Milton, Jennifer McIntosh, Lucy Boyd, Napin Karnchanachari, Finlay Macrae, Jon David Emery

**Affiliations:** 1 Centre for Cancer Research, University of Melbourne, Level 10, 305 Grattan Street, Melbourne, VIC 3000 Australia; 2 Department of General Practice, University of Melbourne, Melbourne, Australia; 3 HumaniSE Lab, Department of Software Systems and Cybersecurity, Monash University, Melbourne, Victoria Australia; 4 Department of Medicine, The University of Melbourne, Melbourne, Australia; 5 Colorectal Medicine and Genetics, The Royal Melbourne Hospital, Melbourne, Australia; 6 The Primary Care Unit, University of Cambridge, Cambridge, UK

**Keywords:** COVID-19, Trial participant recruitment, General practice, Primary care, Cancer prevention, Bowel cancer, Colorectal cancer

## Abstract

**Background:**

Many non-COVID-19 trials were disrupted in 2020 and either struggled to recruit participants or stopped recruiting altogether. In December 2019, just before the pandemic, we were awarded a grant to conduct a randomised controlled trial, the Should I Take Aspirin? (SITA) trial, in Victoria, the Australian state most heavily affected by COVID-19 during 2020.

**Main body:**

We originally modelled the SITA trial recruitment method on previous trials where participants were approached and recruited in general practice waiting rooms. COVID-19 changed the way general practices worked, with a significant increase in telehealth consultations and restrictions on in person waiting room attendance. This prompted us to adapt our recruitment methods to this new environment to reduce potential risk to participants and staff, whilst minimising any recruitment bias. We designed a novel teletrial model, which involved calling participants prior to their general practitioner appointments to check their eligibility. We delivered the trial both virtually and face-to-face with similar overall recruitment rates to our previous studies.

**Conclusion:**

We developed an effective teletrial model which allowed us to complete recruitment at a high rate. The teletrial model is now being used in our other primary care trials as we continue to face the impacts of the COVID-19 pandemic.

## Background

To understand the breadth of research being conducted globally, as of May 2020, ClinicalTrials.gov listed 351,526 ongoing randomised clinical trials, including 98,051 trials of behavioural interventions [1]. In response to the COVID-19 pandemic about 80% of non-COVID-19 trials were interrupted for several reasons including; laboratories closing, a shutdown in communication and because they were unable to be conducted whilst ensuring recruitment safety and efficacy [2].

In December 2019, we were awarded a Victorian Cancer Agency grant to conduct the *Should I Take Aspirin?* (SITA) trial, a randomised controlled trial of a decision aid to support informed choices about taking aspirin to reduce risk of bowel cancer and other chronic conditions [3]. One month later in January 2020, the first case of COVID-19 was detected in Australia, and by the end of March, a national lockdown was in place [4]. The Australian state of Victoria, where our study is being conducted, has been most heavily impacted by COVID-19 compared to the rest of the country. As of the 9 June 2021, Victoria, and mostly in Melbourne, recorded 20,650 out of 30,204 total cases of COVID-19 and 820 out of 910 deaths due to COVID-19 in Australia [5, 6].

To stop the spread of COVID-19 in Australia, lockdowns have been put in place which included varying degrees of self-isolation, limited travel, border closures and only leaving home for daily exercise, buying essentials, authorised work and medical care including getting the COVID-19 test or vaccination [7]. In response to the lockdown, there was a 30% decline in face-to-face general practitioner (GP) appointments visits, which has persisted, and a compensatory increase in telehealth consultations [8, 9]. Consequently, commencing on 13 March 2020, new Medicare Benefits Schedule (MBS) telehealth item numbers were created to allow GP consultations over the phone to be charged to the MBS, Australia’s national healthcare scheme [10]. We had to quickly adapt our trial to ensure successful recruitment and thus completion of the trial, whilst accounting for the shift from face to face consultations to consulting by telehealth.

### Recruiting patients from general practice clinics before COVID-19

In our previous studies, participants were approached and recruited in general practice waiting rooms [11–16]. One of our studies, the *Colorectal cancer RISk Prediction* (CRISP) trial [17], which commenced in 2017, recruited patients in general practice waiting rooms. This method proved to be effective for patient level recruitment and randomisation, thus we planned to model SITA’s recruitment strategy after CRISP.

The recruitment method required two research assistants (RAs) to be in the clinic at the same time; RA1 would approach patients in the waiting rooms whilst RA2 would deliver the trial intervention. Each day in the general practices, RA1 would coordinate with the practice manager and waiting room staff about potentially eligible patients with GP appointments. RA1 would approach patients in the waiting rooms and invite them into the trial. Only participants whose GPs were running late by at least 15 min were approached.

RA1 would then take the patient to a private consultation room with RA2. RA2 would check the participant’s eligibility, gain informed consent, complete the baseline questionnaire, randomise them and then deliver the intervention, a risk assessment tool or control brochure to the participant. RA1 would continue to approach patients in the waiting room whilst ensuring that if the GP called the patient, the patient would be taken to the GP appointment, regardless of whether the research consultation was interrupted or not. From March 2017 to March 2018, with two RA teams recruiting simultaneously, the study successfully recruited 734 participants, the full sample size (65% of eligible participants) into the trial, from 10 general practices.

### Recruiting patients from general practice clinics after COVID-19

COVID-19 meant fewer patients were attending practices and those who did were social distanced in waiting rooms and often sat in their car until their appointment. Our waiting room method of recruitment had to change. We quickly pivoted and developed a novel teletrial model for recruitment and delivery of the interventions [3]. This involved calling patients who were scheduled for a general practitioner appointment and checking their eligibility over the phone. GPs gave us consent to call patients from their practice, making it clear as part of the telephone script that the research team were working with the patient’s general practice as part of the trial. We used the consenting GPs scheduled appointment list for the day and following day to identify people who were 50–70 years old and applied the eligibility criteria over the phone. If participants were eligible and interested, they were scheduled for a either a Zoom [18] or face-to-face consultation, depending on their appointment type with their GP, with RA2 10 to 15 min prior to their scheduled GP appointment, depending on the clinic and length of GP appointment time, and whether they were participating via Zoom. Whilst RAs worked in the clinics, masks were worn at all times, the consulting rooms were disinfected and social distancing measures were followed.

Participants who participated via Zoom consented electronically whilst face-to-face participants were given a new pen, to reduce transmission of COVID-19 and consented on paper [3]. We developed trial recruitment materials into a format suitable for sending via email and/or through the post. We also developed videos that presented the decision aid and control brochures. These videos were presented via the Zoom teletrial consultation through the screensharing function, and in the face-to-face research consultations, which ensured that the decision aids were delivered in a standardised manner [3]. The video links can be found below.

## Results

We have recently completed trial recruitment, and over a 6-month period, we successfully randomised 264 participants into the trial, our full sample size, with a recruitment rate of 87% of eligible participants. 92.8% of our participants were approached over the phone, and 20.8% were consented and received the interventions via a Zoom consultation. The remainder elected to have their GP consultation in person and so also had a face-to-face trial consultation. The 8.2% of our participants who were approached face-to-face were also phoned in advance, but could not be contacted, so they were approached in the general practice waiting room. The teletrial recruitment rate was higher to that achieved in our CRISP trial.

## Conclusion

We have developed an effective teletrial model for the SITA trial which has allowed us to continue during the COVID-19 pandemic and provide more flexible participation options for participants, at a higher rate than the CRISP trial. In so doing, we may have further minimised potential recruitment biases inherent in our previous methods. We are now applying the teletrial method to other trials in general practice including beyond COVID-19 as an effective approach to increase participation and improve the external validity of our research.

## Video links

Control video: https://youtu.be/BzGHxV4-Yw0

Video decision aid for males: https://youtu.be/p_Ey908EApE

Video decision aid for females: https://youtu.be/cDf_3mlJRoU

## Data Availability

Not applicable.

## Abbreviations

GP: General practitioner
RA: Research assistant
SITA: Should I Take Aspirin?
CRISP: Colorectal cancer RISk Prediction
MBS: Medicare Benefits Schedule
